# Cardiovascular Safety Signals of Oral Versus Topical Minoxidil in FAERS: A Disproportionality Analysis (Analytic Cohort 2012–2025)

**DOI:** 10.3390/life16030522

**Published:** 2026-03-21

**Authors:** Hima Bindu Makkena, Vikas Kasu

**Affiliations:** Independent Researcher, McKinney, TX 75071, USA

**Keywords:** minoxidil, oral administration, topical administration, pharmacovigilance, FAERS, disproportionality analysis

## Abstract

Oral minoxidil, including low-dose regimens, is increasingly used off-label for alopecia, but cardiovascular safety remains a clinical concern. We compared cardiovascular adverse event reporting patterns for oral versus topical minoxidil using a disproportionality analysis of the FDA Adverse Event Reporting System (FAERS). FAERS data (2004Q1–2025Q3) were imported and deduplicated; minoxidil reports were restricted to primary/secondary suspect (PS/SS) drugs and eligible reports from 2012 to 2025. Exposure was classified as ORAL, TOPICAL, BOTH, or UNKNOWN using a standardized route/dose-form dictionary. Signals for Core and Expanded cardiovascular MedDRA Preferred Terms (PTs) were assessed using reporting odds ratios (RORs) with 95% confidence intervals; sensitivity analyses included alopecia-restricted cohorts excluding hypertension indications. In the primary ORAL-versus-TOPICAL cohort (559 oral; 56,947 topical), 23 Core-list PTs and 31 Expanded-list PTs met the signal definition. Strongest primary signals included pericardial effusion (ROR 307; 95% CI 158–597), hypertensive crisis (ROR 1037; 95% CI 133–8117), pulmonary hypertension (ROR 932; 95% CI 118–7368), and pulmonary edema (ROR 1965; 95% CI 114–33,813). In an alopecia-restricted sensitivity cohort excluding hypertension/blood-pressure indications (146 oral; 24,367 topical), hemodynamic and effusion-related PTs (e.g., tachycardia, palpitations, orthostatic hypotension, syncope, and pericardial effusion) remained disproportionately reported, although event counts were smaller and confidence intervals were wider. Oral minoxidil PS/SS reports in FAERS showed disproportionate reporting of several cardiovascular PTs relative to topical minoxidil reports. However, because FAERS is a spontaneous reporting system without exposed-patient denominators and with important limitations including under-reporting, stimulated reporting, incomplete clinical information, and residual confounding, these findings should be interpreted strictly as hypothesis-generating reporting signals rather than evidence of incidence, relative risk, or definitive comparative clinical safety.

## 1. Introduction

Topical minoxidil is widely used for androgenetic alopecia, while low-dose oral minoxidil is increasingly prescribed off-label in dermatologic practice for hair disorders, including androgenetic alopecia and other alopecia subtypes [1,2]. This expanding use has been accompanied by ongoing clinical debate regarding cardiovascular safety. On one hand, dermatology reports and case series have generally described low-dose oral minoxidil as reasonably well tolerated in selected patients, with adverse effects such as hypertrichosis, tachycardia, edema, lightheadedness, and fluid retention reported variably across studies [1,2]. On the other hand, oral minoxidil is a systemic vasodilator originally developed as an antihypertensive agent, and its known pharmacology raises concern regarding hemodynamic and fluid-retention-related adverse effects, including hypotension, tachycardia, edema, and pericardial effusion [3]. In clinical practice, minoxidil is only one component of alopecia management, which may also include other medical, procedural, or device-based approaches depending on alopecia subtype and patient context.

Spontaneous reporting systems can support early pharmacovigilance signal detection, but they cannot estimate incidence or establish comparative clinical risk. While recent studies have characterized safety signals of oral minoxidil alone [4], less is known about how cardiovascular reporting patterns for oral minoxidil compare with those for topical minoxidil within the same spontaneous reporting system. However, oral and topical minoxidil are often used in substantially different clinical contexts: oral minoxidil has historical antihypertensive use and may be reported in patients with greater cardiovascular comorbidity burden, whereas topical minoxidil is used predominantly for alopecia and may reflect a different baseline risk profile. We therefore conducted a formulation-based disproportionality analysis of oral versus topical minoxidil cardiovascular reporting patterns in FAERS to contextualize reporting signals, while recognizing that this comparison is limited by substantial confounding by indication, dose heterogeneity, possible exposure misclassification, and the exploratory nature of spontaneous reporting data.

## 2. Materials and Methods

### 2.1. Data Source and Study Period

Public FAERS quarterly ASCII data files from 2004Q1 through 2025Q3 were downloaded and imported from the U.S. Food and Drug Administration (FDA, Silver Spring, MD, USA) [5]. FAERS contains spontaneous adverse event reports submitted to the U.S. Food and Drug Administration (FDA) from manufacturers, health care professionals, and consumers [6]. After applying prespecified inclusion criteria (minoxidil listed as Primary Suspect or Secondary Suspect, deduplicated to the latest case version, and exposure route classified as oral, topical, or unknown), the eligible analytic minoxidil cohort spanned report years 2012–2025. In our imported FAERS data, only a small number of reports listed minoxidil as a PS/SS drug before 2012; because counts were too sparse for stable disproportionality estimation, we defined the analytic cohort as 2012–2025.

### 2.2. Deduplication and Unit of Analysis

To reduce duplicate counting from follow-up submissions, cases were deduplicated to the most recent version per case identifier, consistent with FDA guidance that the latest case version represents the most current information [6].

### 2.3. Case Identification and Exposure Classification

Reports listing minoxidil as a Primary Suspect (PS) or Secondary Suspect (SS) drug were included. A standardized route/dose-form dictionary was applied to classify each report into one of four mutually exclusive exposure groups: ORAL, TOPICAL, BOTH, or UNKNOWN. Primary analyses compared ORAL versus TOPICAL reports; reports classified as BOTH (*n* = 113) or UNKNOWN (*n* = 3545) were excluded from the primary formulation-based disproportionality analyses and are reported descriptively (counts only) because of potential route misclassification. Dose information in FAERS was frequently missing, inconsistently recorded, non-standardized, or insufficiently reliable for formal dose-based stratification. Accordingly, the ORAL group should be interpreted as a heterogeneous oral minoxidil exposure category rather than as verified low-dose oral minoxidil. This category may include reports from different dosing contexts, including historically higher-dose antihypertensive use, lower-dose dermatologic use for alopecia, and oral exposures with incompletely documented dosing. As a result, our analyses compare oral versus topical reporting patterns as captured in FAERS but cannot isolate dose-specific risk or distinguish low-dose dermatologic oral minoxidil from higher-dose systemic use.

- ORAL: at least one oral minoxidil product recorded in the report and no topical minoxidil product recorded.
- TOPICAL: at least one topical minoxidil product recorded in the report and no oral minoxidil product recorded.
- BOTH: evidence of both oral and topical minoxidil products within the same report (possible dual exposure or conflicting entries).
- UNKNOWN: route could not be assigned due to missing/blank route, non-informative dose form, or conflicting route versus dose-form evidence; an unknown_reason field was retained for auditing.

This rule-based classification approach was designed to maximize transparency and reproducibility, but it was not formally validated against an external gold standard or manual chart-level review. Because FAERS drug route and formulation fields may be incomplete, inconsistent, or conflicting, some exposure misclassification is possible. To reduce this risk in the primary comparison, we restricted the main disproportionality analyses to ORAL versus TOPICAL reports only and excluded BOTH and UNKNOWN classifications from primary modeling while retaining them descriptively for transparency. However, this exclusion may also introduce selection bias if reports assigned to BOTH or UNKNOWN differ systematically from those retained in the primary analysis. Accordingly, excluding these categories likely represents a tradeoff between reducing exposure misclassification and potentially altering the analytic population in a non-random way; residual misclassification and selection bias may therefore both influence the observed disproportionality estimates.

### 2.4. Outcomes

Cardiovascular outcomes were defined a priori using MedDRA (Medical Dictionary for Regulatory Activities) Preferred Terms (PTs) from the REAC table, coded using MedDRA version 28.1 (MedDRA Maintenance and Support Services Organization, McLean, VA, USA) [7]. The primary outcome set was a Core cardiovascular PT list, and a broader Expanded list (Core plus edema/volume-related PTs and dizziness) was used for sensitivity analyses.

### 2.5. Statistical Analysis

Disproportionality was assessed using reporting odds ratios (RORs) with 95% confidence intervals (CIs) for each prespecified MedDRA preferred term (PT), comparing ORAL versus TOPICAL minoxidil reports [8,9]. Signal detection required at least 3 oral events for the PT, ROR greater than 1, and a lower 95% CI greater than 1. When a cell count was zero, a standard continuity correction was applied to permit estimation [10,11]. Because the primary cohort was highly imbalanced in size (559 ORAL reports versus 56,947 TOPICAL reports), ROR estimates were interpreted cautiously, particularly for PTs with small oral event counts and very low comparator counts in the topical group. Under these conditions, disproportionality estimates may become unstable and numerically large, with wide confidence intervals, and especially extreme values may reflect sparse-data behavior and continuity-correction effects rather than a clinically precise measure of comparative reporting magnitude. Core and Expanded cardiovascular PT lists were prespecified a priori to reduce data-driven outcome selection. However, no formal statistical adjustment for multiple testing and no empirical Bayesian shrinkage approach were applied in the present analysis. Accordingly, the identified signals should be interpreted as hypothesis-generating pharmacovigilance observations, and some may represent chance findings arising from evaluation of multiple PTs rather than direct measures of clinical effect size or comparative risk. As an exploratory descriptive analysis, yearly counts of oral minoxidil PS/SS reports were summarized by report year (derived from FDA_DT) to characterize temporal reporting patterns.

### 2.6. Sensitivity Analyses

Two prespecified sensitivity analyses were performed. First, we analyzed the Expanded cardiovascular PT list in addition to the Core list primary analysis. Second, to partially explore confounding by indication, we conducted an indication-restricted analysis limited to reports containing alopecia-related indication terms and excluding reports with hypertension/blood-pressure-related indication terms; within this restricted cohort, Core and Expanded PT analyses were repeated. This sensitivity analysis was intended to reduce, but not eliminate, differences in prescribing context between oral and topical minoxidil reports, because FAERS does not provide the detailed clinical information needed to balance groups on comorbidity burden, disease severity, concomitant therapies, baseline cardiovascular risk, or reliably recorded dose. Accordingly, even within the alopecia-restricted cohort, the oral category should not be interpreted as a uniformly low-dose dermatologic exposure group.

### 2.7. Reproducibility

FAERS is publicly available from the U.S. Food and Drug Administration (FDA, Silver Spring, MD, USA). All cohort construction and analyses were generated programmatically from FAERS ASCII files (2004Q1–2025Q3) using version-controlled scripts in Python version 3.13.3 and Pg Admin 4 version 9.11. A public GitHub repository (with a step-by-step README) provides the full workflow used in this study, including FAERS import, deduplication, minoxidil exposure classification, cohort construction, signal generation, and production of manuscript tables and figures. The repository also documents the rule-based exposure-classification logic used to assign reports to ORAL, TOPICAL, BOTH, or UNKNOWN categories, including auditable handling of unknown-route reasons. In addition, the reproducibility materials describe the computational environment, software dependencies, script order, expected inputs and outputs, and the sequence of analytic steps required to regenerate the reported results from the raw FAERS files. Although this documentation supports reproducibility and auditability of the workflow, it should not be interpreted as formal external validation of the exposure-classification algorithm. Repository: https://github.com/vikaskasu92/faers-minoxidil-cv-analysis (accessed on 17 March 2026).

## 3. Results

### 3.1. Study Population

The primary oral versus topical cohort included 559 oral and 56,947 topical minoxidil suspect reports (PS/SS). Baseline characteristics are summarized in Table 1. Eligible PS/SS minoxidil reports with classified exposure route were observed from 2012 onward.

A brief analysis of temporal reporting trends for oral minoxidil revealed fluctuations over the 14-year study period, with notable reporting peaks occurring in 2019 (n = 88) and 2022 (n = 81). Consistent with a recent surge in clinical and public interest, a substantial proportion of the oral minoxidil cohort—239 reports (42.8%)—were submitted from 2022 onward.

### 3.2. Primary Disproportionality Analysis: Core Cardiovascular PT List

**Figure 1:**
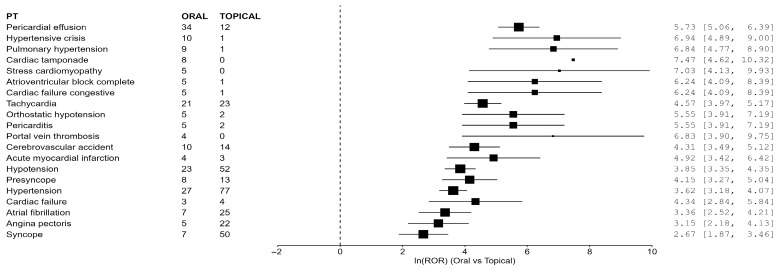
Forest plot of top 20 Core cardiovascular PT signals (log ROR scale).

**Figure 2:**
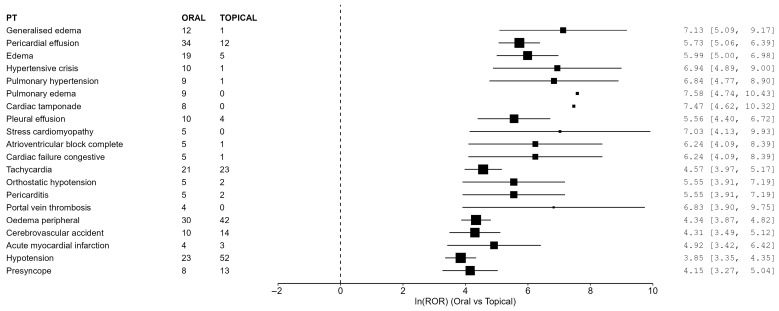
Forest plot of top 20 Expanded cardiovascular PT signals (log ROR scale).

In the primary ORAL-versus-TOPICAL cohort, 559 oral and 56,947 topical reports were included. Using the prespecified Core cardiovascular PT list, 23 PTs met the signal definition (Table 2; Figure 1). Using the Expanded PT list, 31 PTs met the signal definition (Table 3; Figure 2). The strongest RORs were observed for several hemodynamic, effusion-related, and edema-related PTs, including pericardial effusion, hypertensive crisis, pulmonary hypertension, and pulmonary edema. However, several of the most extreme estimates arose in the setting of small oral event counts and extremely low comparator counts in the topical group, including some PTs with zero topical events requiring continuity correction. These values were also accompanied by wide confidence intervals. In addition, because multiple PTs were evaluated across prespecified Core and Expanded lists without formal multiplicity adjustment, some observed signals may reflect chance findings. Therefore, the magnitude and statistical significance of these estimates should be interpreted cautiously, and the findings are best viewed as signals of disproportionate reporting within FAERS rather than clinically confirmatory evidence of comparative risk.

### 3.3. Sensitivity Analysis: Expanded Cardiovascular PT List

In the Expanded PT list sensitivity analysis, 31 PTs met the prespecified signal definition. Edema and fluid-retention PTs (e.g., generalized edema, edema, pulmonary edema, peripheral edema) were prominent in this broader list, and dizziness was included a priori in the Expanded PT set. Table 3 and Figure 2 present the top 20 Expanded PT signals by lower confidence bound.

**Table 3:** Expanded cardiovascular PT safety signals (top 20 shown; ORAL versus TOPICAL, all-cohort primary analysis).

Preferred Term (PT)	Oral Events/Denom	Topical Events/Denom	ROR (95% CI)
Generalized edema	12/559	1/56,947	1249.27 (162.15–9624.68)
Pericardial effusion	34/559	12/56,947	307.27 (158.22–596.71)
Edema	19/559	5/56,947	400.70 (149.07–1077.07)
Hypertensive crisis	10/559	1/56,947	1037.27 (132.55–8117.10)
Pulmonary hypertension	9/559	1/56,947	931.84 (117.85–7367.93)
Pulmonary edema	9/559	0/56,947	1965.49 (114.25–33,813.06)
Cardiac tamponade	8/559	0/56,947	1755.41 (101.19–30,452.04)
Pleural effusion	10/559	4/56,947	259.30 (81.08–829.31)
Stress cardiomyopathy	5/559	0/56,947	1129.71 (62.39–20,456.04)
Atrioventricular block complete	5/559	1/56,947	513.95 (59.95–4406.49)
Cardiac failure congestive	5/559	1/56,947	513.95 (59.95–4406.49)
Tachycardia	21/559	23/56,947	96.61 (53.14–175.61)
Orthostatic hypotension	5/559	2/56,947	256.97 (49.75–1327.37)
Pericarditis	5/559	2/56,947	256.97 (49.75–1327.37)
Portal vein thrombosis	4/559	0/56,947	922.64 (49.61–17,158.20)
Edema peripheral	30/559	42/56,947	76.84 (47.72–123.71)
Cerebrovascular accident	10/559	14/56,947	74.07 (32.76–167.49)
Acute myocardial infarction	4/559	3/56,947	136.80 (30.55–612.68)
Hypotension	23/559	52/56,947	46.95 (28.53–77.26)
Presyncope	8/559	13/56,947	63.59 (26.25–154.03)

Notes: Expanded list includes the Core PT set plus edema/volume-related PTs and dizziness (prespecified). Signal definition as in Table 2.

### 3.4. Patient Outcomes (OUTC) Summary

Patient outcome fields were frequently missing, especially among topical reports (Table 4). OUTC categories are therefore presented for descriptive context only. Although recorded death, hospitalization, and other seriousness outcomes were tabulated for oral and topical reports, these values should not be interpreted as comparative indicators of clinical severity between formulations. In particular, the very high proportion of missing outcome information in the topical group makes direct oral-versus-topical comparison of seriousness categories unreliable and potentially misleading. Apparent differences in recorded hospitalization or death outcomes may reflect differential outcome capture, reporting practices, or missingness patterns rather than true differences in clinical severity. Accordingly, OUTC findings in this study are descriptive only and should not be interpreted as comparative severity estimates.

### 3.5. Indication-Based Sensitivity Analysis

To partially explore confounding by indication, we restricted the cohort to reports with alopecia-related indication PTs and excluded reports with hypertension/blood-pressure-related indication PTs. This sensitivity cohort included 146 oral and 24,367 topical reports. In this restricted cohort, 10 Core PTs met the prespecified signal definition (Supplementary Table S1; Supplementary Figure S1). Hemodynamic and effusion-related PTs remained prominent in the restricted analysis, and Expanded-list analyses identified additional edema/fluid-retention PTs while retaining key hemodynamic signals (Supplementary Table S2; Supplementary Figure S2). However, these restricted analyses should be interpreted cautiously. Although exclusion of hypertension/blood-pressure indications reduced one important source of confounding, oral and topical minoxidil reports in FAERS may still differ in comorbidity burden, prescribing context, co-medications, baseline cardiovascular risk, and dose distribution in ways that cannot be adequately adjusted for within FAERS. In addition, the oral subgroup in the restricted cohort was substantially smaller, making sparse-data behavior even more influential for rare PTs. Because multiple PTs were also examined in these sensitivity analyses without formal multiplicity adjustment, some identified signals may represent chance findings. Accordingly, persistence of signals in the restricted cohort should be interpreted as supportive of signal robustness within FAERS rather than as evidence that confounding, dose heterogeneity, statistical instability, or false-positive risk have been resolved.

## 4. Discussion

In this FAERS disproportionality analysis restricted to minoxidil primary/secondary suspect reports, oral minoxidil showed disproportionate reporting of multiple cardiovascular PTs compared with topical minoxidil. Signals were strongest for pericardial effusion, hypertensive crisis, pulmonary hypertension, and hypotension-related PTs in the Core list, and edema/fluid-retention PTs emerged in the Expanded list. The prominence of effusion and fluid-retention signals is biologically plausible given minoxidil’s systemic hemodynamic effects and labeling that highlights fluid retention and pericardial effusion risk [3]. Pericardial effusion has also been described in recent alopecia-focused reports and series of low-dose oral minoxidil, supporting external validity [12,13]. In addition, severe adverse events related to compounding errors have been reported, underscoring the importance of accurate dosing and monitoring [14]. An editorial commentary has also highlighted pericardial effusion as a clinically relevant concern with low-dose oral minoxidil [15]. Recent reviews synthesize a generally favorable benefit-risk profile for low-dose oral minoxidil while emphasizing patient selection and follow-up [1,16].

Unlike Gupta et al. [4], which evaluated oral minoxidil in FAERS without a topical comparator, our study used topical minoxidil as a formulation-based reference group to contextualize cardiovascular reporting disproportionality for oral minoxidil within the same spontaneous reporting system. We further prespecified Core and Expanded cardiovascular PT lists, included indication-restricted sensitivity cohorts, and analyzed a longer FAERS time horizon. However, the oral-versus-topical comparison has important interpretive limitations. Oral minoxidil has historical antihypertensive use and may be reported in patients with greater baseline cardiovascular comorbidity, different disease severity, or different concomitant medication exposure than topical minoxidil users, who are more often treated for alopecia in otherwise healthier outpatient settings. In addition, reliable dose stratification was not possible in FAERS because dosage information was frequently missing, incomplete, inconsistently recorded, or non-standardized. As a result, the oral category in this study likely includes a mixture of higher-dose antihypertensive use, lower-dose dermatologic use, and oral exposures with unclear dosing. Because FAERS does not contain sufficient clinical detail to balance or adjust for these differences, the oral and topical groups should not be considered clinically exchangeable comparator populations, and the oral category should not be interpreted as a verified low-dose oral minoxidil cohort. Our alopecia-restricted, no-hypertension-indication sensitivity analysis was therefore intended only to partially reduce one major source of confounding by indication. The persistence of several hemodynamic and effusion-related reporting signals in that restricted cohort may support the consistency of those reporting patterns within FAERS, but it does not eliminate residual confounding or dose heterogeneity, nor does it establish definitive comparative safety differences or confirm specific mechanistic associations between minoxidil exposure and the observed events. Rather, these findings remain hypothesis-generating and should be interpreted as signals that may be compatible with known pharmacologic effects of minoxidil, while still requiring confirmation in more rigorously controlled data sources.

Thrombotic PTs observed with very small counts (e.g., portal vein thrombosis in the alopecia-restricted cohort) should be interpreted with particular caution. These findings arose from sparse event counts and may be influenced by residual confounding, coexisting comorbidities, concomitant therapies, and statistical instability. Although patients with alopecia areata may in some settings receive medications such as Janus kinase (JAK) inhibitors that have recognized thrombotic safety considerations in other contexts [17,18,19], we did not directly analyze co-medications, treatment combinations, or drug–drug interactions within the FAERS reports in this study. Accordingly, any explanation invoking concomitant JAK inhibitor use remains speculative. These rare thrombotic observations should therefore be regarded strictly as hypothesis-generating signals of uncertain attribution rather than as evidence of a specific co-medication effect or a confirmed minoxidil-associated thrombotic risk pattern.

Interpretation of seriousness outcomes (death, hospitalization) is limited by substantial outcome missingness, particularly among topical reports, and these OUTC fields should therefore be treated strictly as descriptive rather than comparative. More generally, FAERS analyses cannot estimate incidence and are subject to under-reporting, stimulated reporting, reporting bias, incomplete clinical detail (including dose and comorbidities), residual confounding, comparator non-exchangeability, false-positive risk when numerous PTs are examined, possible exposure misclassification, and selection effects arising from analytic inclusion criteria. In practical terms, this means that the present findings should not be used to infer how often these cardiovascular events occur in routine clinical use, to rank the absolute clinical safety of oral versus topical minoxidil, or to make direct treatment decisions based on effect size estimates alone. Instead, the signals are most appropriately interpreted as indicators of which cardiovascular events may warrant greater clinical awareness and further evaluation in better-controlled data sources. In addition, the marked imbalance between the oral and topical cohorts (559 versus 56,947 reports in the primary analysis) is an important source of interpretive uncertainty. When oral event counts are small and comparator counts in the topical group are very low, disproportionality estimates can become numerically extreme, especially when continuity correction is required for zero cells, and such values may reflect sparse-data behavior or statistical artifacts rather than a clinically stable estimate of comparative reporting magnitude. This concern is particularly relevant for PTs with very large RORs and wide confidence intervals. Although our Core and Expanded PT lists were prespecified to reduce data-driven outcome selection and a stringent signal definition was used, no formal false discovery rate correction or empirical Bayesian approach was applied. Therefore, some observed signals may represent chance findings in addition to sparse-data instability. The high RORs may also partially reflect Weber-like or stimulated reporting effects for the oral formulation due to renewed public and clinical attention compared with longstanding familiarity with the topical formulation [20,21]. Because exposure classification relied on a practical rule-based dictionary applied to incomplete FAERS route/formulation fields and was not formally externally validated, some oral/topical misclassification may remain despite exclusion of BOTH and UNKNOWN reports from the primary comparison. Excluding BOTH and UNKNOWN reports likely reduced one source of exposure ambiguity, but it may also have altered the analytic population if excluded reports differed systematically from included reports; thus, selection bias in addition to misclassification bias remains possible. Known pharmacologic properties of minoxidil, including vasodilation and fluid-retention effects, may provide biologic plausibility for some observed reporting patterns; however, such plausibility should not be interpreted as confirmation that the reported FAERS signals represent causal or mechanistically established drug effects in this dataset. Similarly, although some rare thrombotic findings could theoretically be influenced by concomitant therapies used in alopecia populations, co-medications and drug–drug interactions were not directly analyzed here, so such explanations remain speculative. Accordingly, these findings should be interpreted as evidence that such events were disproportionately reported in FAERS, not as estimates of comparative mortality, hospitalization, incidence, relative risk, clinical severity, precise effect size, confirmed mechanism, or confirmed co-medication interaction. Although the persistence of hemodynamic and effusion-related signals in the alopecia-restricted analysis may support the robustness of those reporting patterns within FAERS, these observations remain hypothesis-generating and should not be interpreted as establishing causal or definitive comparative safety differences between oral and topical minoxidil. Rather, they highlight the need for confirmation in study designs with better characterization of dose, indication, comorbidity burden, exposed-patient denominators, formal control of multiplicity, validated exposure ascertainment, and direct evaluation of concomitant therapies.

### Limitations

First, FAERS is a spontaneous reporting system, and disproportionality measures reflect reporting patterns rather than true incidence. Such analyses are susceptible to under-reporting, stimulated reporting, Weber-like reporting effects [20,21], duplicate reporting, and incomplete or inconsistently documented clinical information, including dose, timing, comorbidities, and co-medications. Because FAERS does not provide reliable denominators for the total number of exposed patients, this study cannot estimate incidence, relative risk, or definitive comparative safety between oral and topical minoxidil. Second, confounding by indication and comparator non-exchangeability are major limitations of this study. Oral minoxidil has historical antihypertensive use and may be reported in patients with greater baseline cardiovascular disease, more severe underlying illness, or different concomitant therapies than topical minoxidil users, who are more often treated for alopecia and may differ substantially in baseline risk profiles. As a result, oral and topical reports in FAERS should not be interpreted as directly comparable clinical populations. Our alopecia-restricted, no-hypertension-indication sensitivity analysis was intended to reduce, but cannot eliminate, this concern; moreover, the oral subgroup in that sensitivity cohort was smaller, yielding wider confidence intervals and reduced precision for rare outcomes. Third, reliable dose stratification was not possible because dose information in FAERS was frequently missing, incompletely recorded, inconsistently formatted, or otherwise insufficient for robust categorization. Consequently, the oral exposure group in this study likely combines reports from different dosing strategies, including historically higher-dose antihypertensive therapy, lower-dose dermatologic use for alopecia, and oral exposures with uncertain dose documentation. These dosing contexts may involve substantially different pharmacologic exposures and underlying risk profiles; therefore, this analysis cannot isolate low-dose oral minoxidil and should not be interpreted as a dose-specific safety assessment. Fourth, the study population was highly imbalanced in size, with 559 oral reports and 56,947 topical reports in the primary cohort. This large disparity may distort disproportionality estimates, particularly for PTs with small oral event counts and very low event counts in the topical comparator group. Under such conditions, RORs can become unstable and numerically extreme, especially when continuity correction is applied for zero cells, and thus may overstate the apparent magnitude of reporting disproportionality. Fifth, although Core and Expanded PT lists were prespecified to reduce data-driven outcome selection, many PTs were evaluated across the primary and sensitivity analyses, and no formal statistical adjustment for multiple testing or empirical Bayesian shrinkage approach was applied. As a result, some observed signals may represent chance findings. Sixth, exposure classification relied on report fields and a rule-based route/dose-form dictionary to assign reports to ORAL, TOPICAL, BOTH, or UNKNOWN categories. Although this approach was transparent and reproducible, it was not formally validated against an external gold standard or manual chart-level review. Because FAERS route and formulation fields may be incomplete, inconsistent, or conflicting, some exposure misclassification is possible. Excluding BOTH and UNKNOWN reports from the primary comparison likely reduced exposure ambiguity, but it may also have introduced selection bias if excluded reports differed systematically from those retained in the ORAL and TOPICAL groups. Thus, the primary analytic cohort may reflect both reduced misclassification and altered sample composition, and either mechanism could influence signal detection and the apparent contrast between formulations. Seventh, outcome fields such as death and hospitalization showed substantial missingness, especially among topical reports. Therefore, OUTC categories in this study should be interpreted strictly as descriptive only. Apparent differences in seriousness outcomes between oral and topical reports may reflect differential outcome capture and missingness patterns rather than true differences in clinical severity. Eighth, although some observed signals may be biologically plausible given the known pharmacology of minoxidil, such plausibility does not establish causality or confirm specific mechanistic associations within a spontaneous reporting dataset. Finally, we did not directly analyze co-medications, treatment combinations, or drug–drug interactions within FAERS reports. Therefore, explanations invoking concomitant therapies—such as JAK inhibitors in alopecia populations—remain speculative and cannot be substantiated from the present analysis. Accordingly, all signals reported here should be interpreted strictly as hypothesis-generating pharmacovigilance observations rather than as proof of causal, mechanistic, dose-specific, co-medication-specific, or comparative clinical risk.

## 5. Conclusions

Oral minoxidil suspect reports in FAERS showed disproportionate reporting of several cardiovascular preferred terms relative to topical minoxidil suspect reports. In sensitivity analyses restricted to alopecia indications and excluding hypertension/blood-pressure indications, several hemodynamic and effusion-related signals remained disproportionately reported, although estimates were less precise because of smaller oral counts. These findings should be interpreted strictly as hypothesis-generating pharmacovigilance signals within a spontaneous reporting system. Because oral and topical minoxidil reports likely arise from clinically different patient populations that cannot be adequately balanced within FAERS, this formulation-based comparison should not be interpreted as a definitive head-to-head comparative safety assessment. In addition, because dose information was frequently missing or inconsistently recorded, the oral category likely represents a heterogeneous mixture of dosing contexts and should not be interpreted as a verified low-dose oral minoxidil cohort. The marked imbalance between the oral and topical cohorts, together with sparse event counts for some PTs, may also contribute to unstable or numerically extreme disproportionality estimates. Moreover, because multiple PTs were evaluated without formal multiplicity adjustment or empirical Bayesian shrinkage, some signals may represent chance findings. Seriousness outcomes such as death and hospitalization were highly incomplete in FAERS, particularly among topical reports, and should therefore be interpreted descriptively rather than comparatively. Although some observed reporting patterns may be compatible with known pharmacologic effects of minoxidil, they do not establish causal or mechanistically confirmed associations, and rare thrombotic observations cannot be specifically attributed to concomitant therapies because co-medications were not directly analyzed. Confirmation in studies with better control of indication, comorbidity burden, dosing, exposed-patient denominators, multiplicity, validated exposure ascertainment, and concomitant medication assessment is needed.

## Figures and Tables

**Table 1:** Demographic and reporting characteristics (deduplicated FAERS cases; oral versus topical minoxidil, PS/SS).

Exposure Group	Denominator	Female	Male	Sex Unknown	Age Missing	Age, Median (IQR)	Age, Mean (SD)	Report Year Range	Report Year Median
ORAL	559	215 (38.46%)	271 (48.48%)	73 (13.06%)	126 (22.54%)	52 (32–65)	46.59 (24.67)	2012–2025	2020
TOPICAL	56,947	24,315 (42.70%)	27,300 (47.94%)	5332 (9.36%)	41,272 (72.47%)	61 (46–71)	58.23 (18.32)	2012–2025	2021

Notes: Age and sex are based on DEMO fields; age statistics exclude missing ages. IQR = interquartile range. Report year derived from FDA_DT. The analytic cohort began in 2012 because only a small number of reports listed minoxidil as a PS/SS drug before 2012 in our import (raw DEMO and deduplicated latest-case dataset), and counts prior to 2012 were too sparse for stable disproportionality estimation.

**Table 2:** Core cardiovascular PT safety signals (top 20 shown; ORAL versus TOPICAL, all-cohort primary analysis).

Preferred Term (PT)	Oral Events/Denom	Topical Events/Denom	ROR (95% CI)
Pericardial effusion	34/559	12/56,947	307.27 (158.22–596.71)
Hypertensive crisis	10/559	1/56,947	1037.27 (132.55–8117.10)
Pulmonary hypertension	9/559	1/56,947	931.84 (117.85–7367.93)
Cardiac tamponade	8/559	0/56,947	1755.41 (101.19–30,452.04)
Stress cardiomyopathy	5/559	0/56,947	1129.71 (62.39–20,456.04)
Atrioventricular block complete	5/559	1/56,947	513.95 (59.95–4406.49)
Cardiac failure congestive	5/559	1/56,947	513.95 (59.95–4406.49)
Tachycardia	21/559	23/56,947	96.61 (53.14–175.61)
Orthostatic hypotension	5/559	2/56,947	256.97 (49.75–1327.37)
Pericarditis	5/559	2/56,947	256.97 (49.75–1327.37)
Portal vein thrombosis	4/559	0/56,947	922.64 (49.61–17,158.20)
Cerebrovascular accident	10/559	14/56,947	74.07 (32.76–167.49)
Acute myocardial infarction	4/559	3/56,947	136.80 (30.55–612.68)
Hypotension	23/559	52/56,947	46.95 (28.53–77.26)
Presyncope	8/559	13/56,947	63.59 (26.25–154.03)
Hypertension	27/559	77/56,947	37.48 (23.98–58.59)
Cardiac failure	3/559	4/56,947	76.81 (17.15–344.01)
Atrial fibrillation	7/559	25/56,947	28.87 (12.44–67.04)
Angina pectoris	5/559	22/56,947	23.35 (8.81–61.89)
Syncope	7/559	50/56,947	14.43 (6.51–31.97)

Notes: ROR = reporting odds ratio. Signals defined as ≥3 oral reports and lower 95% CI > 1. Continuity correction (add 0.5 to all four cells) applied when any cell count was zero.

**Table 4:** FAERS patient outcomes (OUTC) by exposure group (oral versus topical).

Exposure Group	Death	Hospitalization	Life-Threatening	Disability	Congenital Anomaly	Required Intervention	Other Serious	BLANK_ONLY	MISSING
ORAL (n = 559)	46 (8.23%)	207 (37.03%)	28 (5.01%)	14 (2.50%)	1 (0.18%)	12 (2.15%)	285 (50.98%)	6 (1.07%)	107 (19.14%)
TOPICAL (n = 56,947)	16 (0.03%)	194 (0.34%)	36 (0.06%)	83 (0.15%)	6 (0.01%)	14 (0.02%)	802 (1.41%)	43 (0.08%)	55,875 (98.12%)

Notes: OUTC categories follow U.S. serious outcome reporting definitions. MISSING indicates no OUTC record for the report; BLANK_ONLY indicates an OUTC record exists but outcome value is blank.

## Data Availability

FAERS data are publicly available from the U.S. Food and Drug Administration. The analytic code and a step-by-step reproducibility README are available in a public repository: https://github.com/vikaskasu92/faers-minoxidil-cv-analysis (accessed on 17 March 2026). Derived, aggregated counts used for tables/figures can be reproduced by re-running the provided scripts against the imported FAERS ASCII data.

## Supplementary Materials

The following supporting information can be downloaded at https://www.mdpi.com/article/10.3390/life16030522/s1: Figure S1 (Forest plot of Core cardiovascular PT signals in the alopecia-restricted/no-hypertension cohort); Table S1 (Core cardiovascular PT signals in the alopecia-restricted/no-hypertension cohort); Figure S2 (Forest plot of Expanded cardiovascular PT signals in the alopecia-restricted/no-hypertension cohort); Table S2 (Expanded cardiovascular PT signals in the alopecia-restricted/no-hypertension cohort).
