# Supplementary material for: Cardiovascular Safety Signals of Oral Versus Topical Minoxidil in FAERS: A Disproportionality Analysis (Analytic Cohort 2012–2025)
Source: Life (Basel). 2026 Mar 21;16(3):522. doi: 10.3390/life16030522 (PMC13028299; doi:10.3390/life16030522)

Supporting Information

Supporting information for “Cardiovascular safety signals of oral versus topical minoxidil in FAERS: a disproportionality analysis (analytic cohort 2012–2025)”.

Table S1. Core cardiovascular PT signals in the alopecia-restricted/no-hypertension cohort

Preferred Term (PT)	Oral events/denom	Topical events/denom	ROR (95% CI)
Orthostatic hypotension	5/146	1/24367	864.04 (100.30–7443.28)
Portal vein thrombosis	4/146	0/24367	1539.00 (82.47–28719.17)
Tachycardia	10/146	10/24367	179.10 (73.36–437.25)
Pericardial effusion	4/146	5/24367	137.25 (36.48–516.42)
Presyncope	4/146	9/24367	76.24 (23.21–250.43)
Angina pectoris	3/146	10/24367	51.10 (13.92–187.62)
Syncope	5/146	32/24367	26.97 (10.36–70.22)
Hypotension	4/146	34/24367	20.16 (7.06–57.56)
Palpitations	9/146	192/24367	8.27 (4.15–16.48)
Chest pain	4/146	122/24367	5.60 (2.04–15.36)

Notes: Sensitivity cohort required an alopecia-related indication PT and excluded hypertension/blood-pressure-related indication PTs.

Table S2. Expanded cardiovascular PT signals in the alopecia-restricted/no-hypertension cohort.

Preferred Term (PT)	Oral events/denom	Topical events/denom	ROR (95% CI)
Generalized edema	6/146	0/24367	2254.64 (126.40–40215.88)
Orthostatic hypotension	5/146	1/24367	864.04 (100.30–7443.28)
Portal vein thrombosis	4/146	0/24367	1539.00 (82.47–28719.17)
Tachycardia	10/146	10/24367	179.10 (73.36–437.25)
Pericardial effusion	4/146	5/24367	137.25 (36.48–516.42)
Edema	3/146	3/24367	170.38 (34.10–851.31)
Presyncope	4/146	9/24367	76.24 (23.21–250.43)
Edema peripheral	8/146	28/24367	50.39 (22.57–112.53)
Angina pectoris	3/146	10/24367	51.10 (13.92–187.62)
Syncope	5/146	32/24367	26.97 (10.36–70.22)
Hypotension	4/146	34/24367	20.16 (7.06–57.56)
Palpitations	9/146	192/24367	8.27 (4.15–16.48)
Chest pain	4/146	122/24367	5.60 (2.04–15.36)
Dizziness	7/146	409/24367	2.95 (1.37–6.34)

Notes: Weight increased did not meet the signal definition (lower 95% CI ≤1) but is listed for completeness.

Supplementary Figure S1. Forest plot of Core PT signals in alopecia-restricted/no-hypertension cohort (log ROR scale).

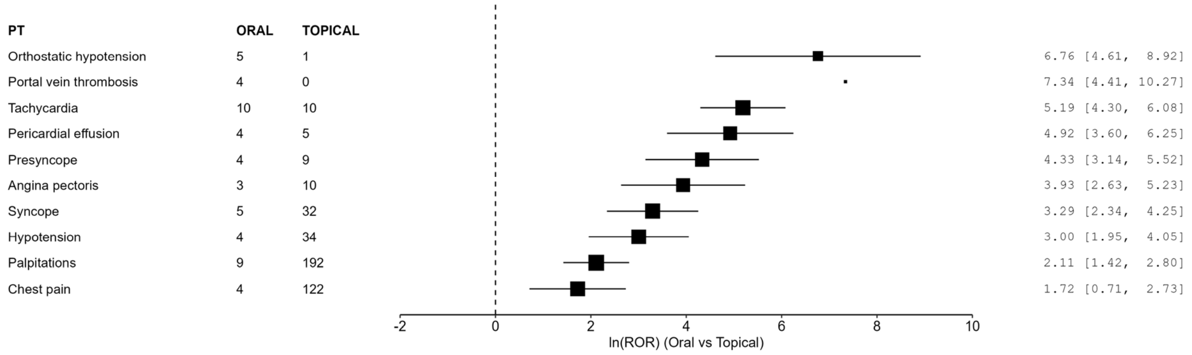

Supplementary Figure S2. Forest plot of Expanded PT signals in alopecia-restricted/no-hypertension cohort (log ROR scale).

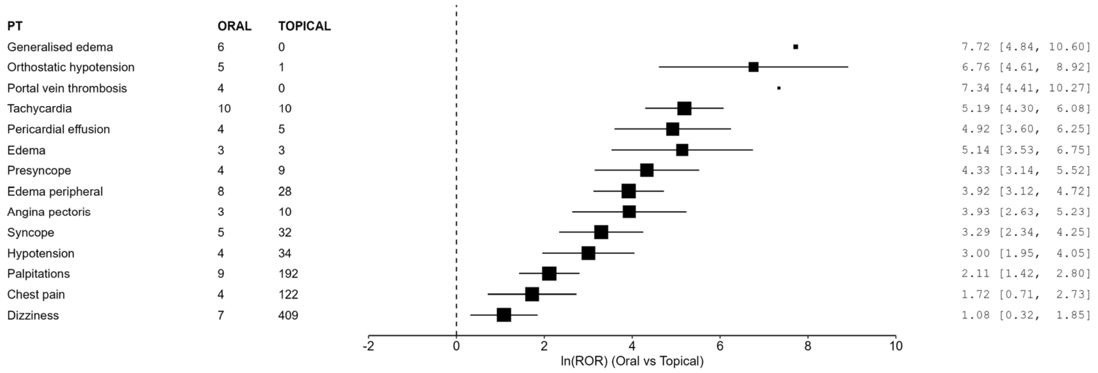

Supplement: Supplementary file 1 [file life-16-00522-s001.zip › life-4199875-supplementary.pdf]
